# Uncontrolled Arterial Bleeding in a Patient With Massive Right Calf Hematoma: A Case Report and a Review of the Literature

**DOI:** 10.7759/cureus.51413

**Published:** 2023-12-31

**Authors:** Yesenia Brito, Bruce W Wilson, Zaid Taki El-Din, Elizabeth Edah, Frederick Tiesenga

**Affiliations:** 1 Surgery, Saint George's University School of Medicine, True Blue, GRD; 2 Psychiatry, Saint George's University School of Medicine, Irvine, USA; 3 Medicine, All Saints University School of Medicine, Roseau, DMA; 4 General Surgery, West Suburban Medical Center, Chicago, USA

**Keywords:** timing of debridement, early debridement, blood disorder, anticoagulants, arterial bleeding, blood thinner, expanding hematoma

## Abstract

A hematoma is a collection of pooled blood that can be confined to a space under the skin, tissue, or organ. It occurs due to injury to the vasculature arising from trauma, previous surgeries, or vascular defects. Anticoagulants can remarkably increase a patient’s risk for hematoma formation. Most hematomas will resolve spontaneously over time, but there are certain instances where surgical intervention becomes necessary.

We present a case of a 71-year-old female on anticoagulants who presented to the emergency department (ED) with an expanding hematoma on her right leg after a fall and had to undergo an emergency surgical evacuation. The etiology, appropriate management, and complications of hematomas will also be covered in this paper.

## Introduction

A hematoma is a localized collection of blood that occurs when blood leaks out of blood vessels and collects in nearby tissues. Many factors, including injuries, trauma, surgery, or underlying medical conditions, can cause a hematoma [1].

Taking an anticoagulant reduces the blood's ability to clot, making it more difficult to stop bleeding and making specific individuals more prone to developing a hematoma. Having a history of cellulitis and deep vein thrombosis (DVT) can also predispose an individual to develop a hematoma as both disrupt the integrity of the blood vessels, which allows blood to collect in tissue [1].

This report highlights the importance of recognizing the risk factors associated with hematoma formation in patients with a history of vascular disorders and on anticoagulants. It emphasizes the need for prompt evaluation and management to prevent further complications.

## Case presentation

A 71-year-old morbidly obese female with a body mass index of 40 presented to the emergency department on February 26, 2023, following a fall. The patient was admitted with right lower leg pain, bruise, and hematoma. She admitted to taking acetaminophen for the pain two hours prior to her ED visit and reported minimal relief. She had been hospitalized for the past two to three months due to an infection in the left foot, which was now resolved. The patient reported erythema on her right leg prior to the fall and denied any head injury, headache, loss of consciousness, nausea, vomiting, fever, and changes in gait. 

The patient's medical history is complex, with a variety of conditions and medical events. In March 2022, she was diagnosed with left lower extremity cellulitis. Following this, in September 2022, the patient underwent surgery for a left foot wound that was found to have osteomyelitis after a bone biopsy was performed. Operative cultures identified the presence of *Peptoniphilus asaccharolyticus *and a gram-negative bacillus (which was not identified further) in the wound along with skin flora.

Additionally, the patient has been managing bilateral leg venous stasis ulcers, which have required multiple debridements between March 2020 and September 2021. Other significant medical events include a diagnosis of DVT in the distal vein of her right lower extremity in 2015, which resulted in a pulmonary embolism. She has also been managing osteoarthritis, anemia, chronic diastolic congestive heart failure, essential hypertension, chronic renal insufficiency, and obstructive sleep apnea.

The patient's current medication regimen included rivaroxaban, simvastatin, spironolactone, and furosemide.

**Figure 1:**
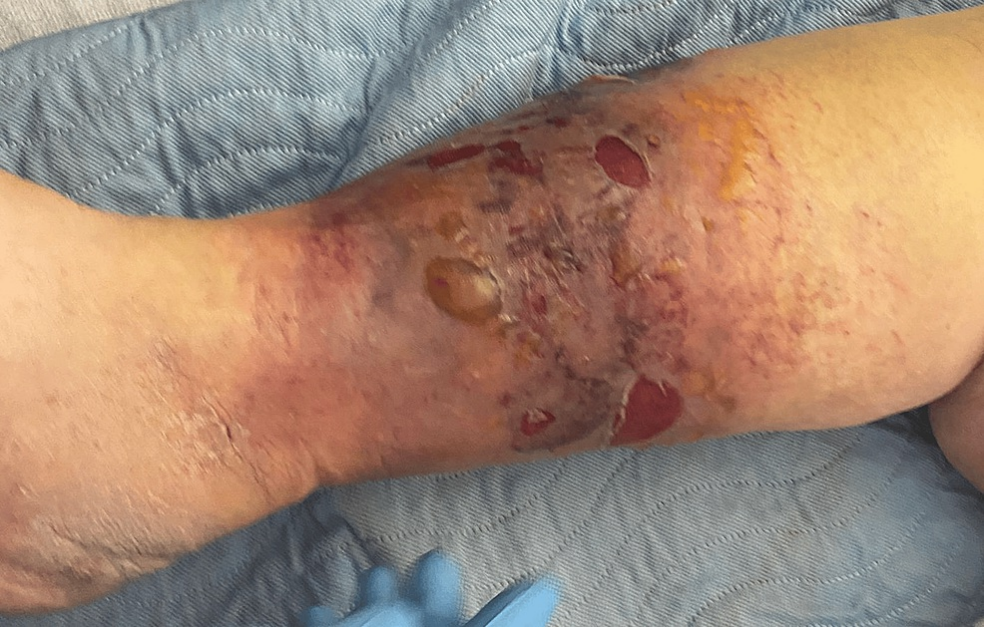
Right lower leg showing several blisters and hyperpigmentation

During the physical exam, the patient was alert and responsive. Her vitals were within the normal limit. The physical exam revealed clear lungs and bilateral leg edema. Several intact blisters appeared on the right lower extremity since her arrival at the ED. The right lower extremity exhibited hyperpigmentation, bullae, and no increased warmth (Figure 1). The patient displayed normal vascular and range of motion indices, along with soft compartments and intact motor and sensory functions. 

The patient's admission laboratory results are shown in Table 1. 

**Table 1:** The patient's laboratory results throughout her stay in the hospital WBC - white blood cell; RBC - red blood cell; MCV - mean corpuscular volume; BUN - blood urea nitrogen

Test	02/26/2023	3/6/2023	3/8/2023	03/20/2023	03/23/2023	Reference range
WBC	15.2	10.5	10.4	9	11.2	4.5–11.0 x 10^9/L
RBC	4	2.44	2.61	3.47	3.35	3.63–5.04 m/mm cu
Hemoglobin	11.2	7.4	7.9	10.1	9.7	12.0–15.5 g/dL
Hematocrit	33.5	21.7	23	30.9	29.2	36.0–46.0%
MCV	83.8	88.8	88.1	89.1	87.4	80.0–100.0 fL
Platelet	231000	304000	376000	432000	355000	150000–450000/uL
Na	140		141	142	139	135–145 mmol/L
K	4.3		4.3	4.2	4.2	3.5–5.0 mmol/L
Cl	105		105	106	103	98–108 mmol/L
CO2	25		29	29	29	22–32 mmol/L
BUN	31		18	14	17	6–20 mg/dL
Creatinine	1.16		0.8	0.7	0.69	0.6–1.3 mg/dL
Glucose	136		95	85	91	70–100 mg/dL
Ca	9		8.2	8.4	8.2	8.5–10.5 mg/dL

**Figure 2:**
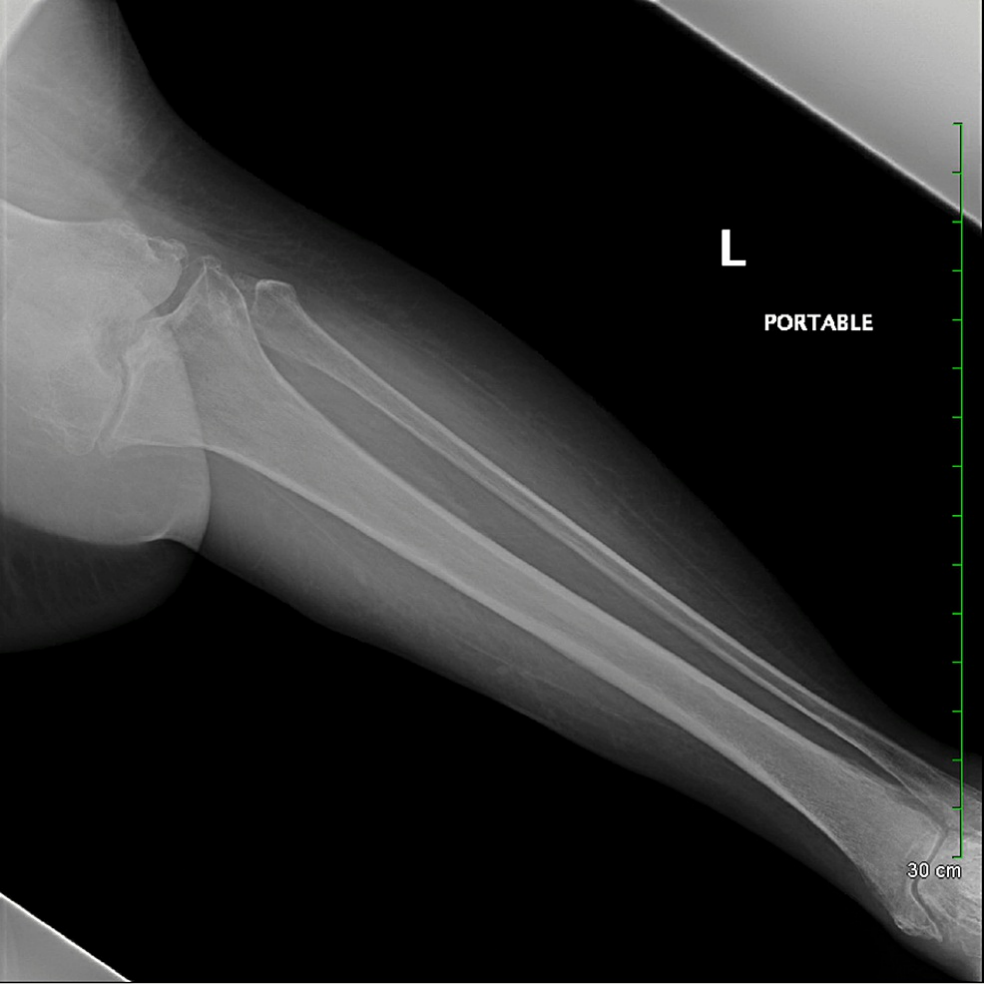
An X-ray of the right tibia and fibula, frontal and lateral views. The bones are demineralized, which is a limiting factor for nondisplaced fracture evaluation. No dominant acute fracture in this long bone study.

The patient was placed on vancomycin and piperacillin/tazobactam. The patient's right leg wound culture and blood cultures were negative. A computer tomography (CT) scan showed no intracranial bleeding. Radiography imaging of the patient's right tibia and fibula revealed osteoarthritis. There was additionally no subcutaneous emphysema, which helped rule out a necrotizing soft tissue infection (Figure 2). A chest radiograph was also performed, which showed no infiltrates.

After one day in the hospital, the patient's lower right leg developed significant swelling as well as a deep reddish/purple discoloration. Large 3-4 cm bullae began developing. No increased warmth was noted. Conservative management was attempted for three days. During this time, the swelling and discoloration did not improve. Bleeding was noted on the posterior calf whenever dressings were changed. At this point, the patient's hemoglobin had decreased from 11.2 g/dL to 7.4 g/dL. After physical therapy during admission, the patient noticed increased bleeding in the lower extremity. Previously, the bleeding had been occasional, but now it was severe enough to soak the patient's sock. 

The surgical team was consulted due to progressive right lower leg bleeding and hematoma. The patient was taken to the operating room where evacuation of a massive right calf hematoma measuring 20 x 40 cm with control of arterial bleeding, skin debridement, and a 10 x 10 cm subcutaneous debridement took place. Antibiotic irrigation was utilized, and several areas of the arterial bleeding were controlled with cautery. At this time, meticulous hemostasis was assured, and the wound was packed open to facilitate proper healing. The surgery was well tolerated by the patient. 

A wound vacuum-assisted closure was ordered and placed on the patient's wound one day after her surgery to promote blood flow to the wound, improve healing, and reduce the risk of infection. 

## Discussion

A hematoma is a localized accumulation of blood beneath the skin, resulting from the disruption of blood vessels [2]. The severity and extent of hematomas can vary, ranging from small, self-limited occurrences to larger, expanding hematomas [3].

In rare cases, a hematoma may present as an expanding hematoma, which is a potentially serious condition. These hematomas contain both old and new blood. The tissue surrounding the expanding hematoma is subject to necrosis and liquefaction. These lesions are most commonly seen in the lower extremities and are usually found in the subcutaneous tissue [4].

Timely intervention is required to reduce the risk of complications. If sufficient blood is lost, patients can become hemodynamically unstable, leading to possible loss of consciousness, acute kidney injury, or even death. Acute compartment syndrome (ACS) should be considered in these patients. While ACS is most commonly seen in younger populations, it has been shown to be associated with anticoagulant use in the elderly [5]. Even if an expanding hematoma does not result in ACS or hemodynamic instability, it can still be dangerous if left untreated, as these hematomas do not self-resolve. If an expanding hematoma remains undrained, it can become a chronic expanding hematoma. These hematomas exhibit an adhesive fibrous capsule around the blood collection. Adhesive fibrous capsules complicate surgical drainage as the capsule should be removed to prevent the recurrence of the hematoma. If the hematoma and capsule are not completely resected, there is a 22% chance of recurrence [4]. Resection becomes more difficult if a hematoma capsule is adjacent to a nerve as they adhere together. In these instances, the capsule should only be partially resected. In one case, the capsule adhered to the common peroneal nerve, and the nerve was not identified. It was accidentally resected, which led to a permanent foot drop in the patient [4]. 

Several risk factors have been identified as potential contributors to hematoma formation. Trauma or injury to the affected area is a common risk factor [2]. The use of anticoagulant or antiplatelet medications can increase the risk of hematoma due to their effects on the blood clotting mechanism [6]. Advanced age has also been recognized as a risk factor as the skin and blood vessels become more fragile and susceptible to injury [7]. Certain blood disorders, such as hemophilia or von Willebrand disease, can impair the body's ability to clot and thereby increase the risk of hematomas [8]. Additionally, underlying vascular conditions, like chronic venous insufficiency, can further emphasize the susceptibility of blood vessels to damage and subsequent hematoma development [9].

Assessment and diagnosis play crucial roles in evaluating and managing hematomas effectively. Clinical assessment involves obtaining a detailed medical history, including information on trauma or underlying medical conditions, and performing a thorough physical examination. Diagnostic imaging techniques, such as ultrasound, CT, or MRI, are commonly employed to aid in the diagnosis and assessment of hematomas [3]. These imaging modalities provide valuable information about the extent of the hematoma, its proximity to vital structures, and any associated complications, which guides appropriate management [3].

A medication review should be performed for a patient. If the patient was taking an anticoagulant, they should be asked about the time of their last dose and if they have any kidney disease. They should also be asked if they are on antiplatelets [10]. Lab work should be ordered to assess the patient's current hemostatic ability. Activated charcoal can help clear direct oral anticoagulants in the case of an overdose or if they were taken within two to three hours of presenting for treatment [10]. Acute kidney injury or poor renal function can delay the clearance of anticoagulants. All patients with a major bleed should have their anticoagulants held, and initial hemostatic therapy, such as compression and direct pressure, should be attempted. If the bleeding does not resolve with these measures, anticoagulant reversal should be considered. Assess the reason the patient was prescribed anticoagulants. In cases of atrial fibrillation or DVT, temporary interruption of anticoagulation usually has no ill effect. In cases with a higher risk of thrombotic events, such as those with mechanical heart valves, consider withholding reversal if possible [10]. If a reversal is required, patients on warfarin should be given prothrombin complex concentrate, patients on dabigatran should be given idarucizumab, and andexanet alpha can be used for patients on factor Xa inhibitors [11].

Most hematomas can be managed conservatively. However, if the blood accumulation is over the shin, it will likely require surgery. If it is in other locations, pressure and elevation of the affected limb can aid in drainage and resolution of the hematoma. If the hematoma does not improve within a week of conservative treatment, and there are skin changes/necrosis or infection, surgical intervention will likely be needed [12]. Hematomas can become inflamed and develop into abscesses that require an emergency incision [13]. Additionally, there may be enlarged regional lymph nodes [13]. If the hematoma is in a critical area, such as the neck, emergency surgical intervention may be required. The variable depth of these hematomas can make expansion rate and volume difficult to estimate. Deep tissue expanding hematomas require less volume to cause significant symptoms. If rapid swelling is noted or there are any signs of a compromised airway, then there should be immediate surgical evaluation. Pressure dressings, ice packs, anticoagulant reversal, and blood pressure normalization can be used to stabilize patients on their way to surgery [14]. In non-critical parts of the body, surgical evaluation should be considered in any case with hemodynamic instability [15].

Tranexamic acid is a coagulation-enhancing drug that is given to treat menorrhagia and prophylactically to hemophilia patients to prevent excessive bleeding during procedures. It is often used off-label in patients with severe bleeding [16]. It is recommended in cases of traumatic bleeding and reduces mortality in these patients. If administered within one hour of injury, tranexamic acid has been shown to reduce mortality by 32% and to reduce mortality by 21% if given within one to three hours of injury [16,17]. However, it is contraindicated in cases of hematuria, as clotting can lead to hydronephrosis [17] and in patients with a history of venous or arterial thromboembolism or active thromboembolic disease [16].

Post-admission medical management is difficult for a patient with a history of major bleeding and clotting events. Our patient has clear indications for anticoagulant therapy, which makes it unlikely for rivaroxaban to be discontinued. Thus, it is useful to study what led to the bleeding and how future events could be prevented. As previously mentioned, this patient fell down the stairs on her way home from a long stay in a nursing facility where she had received treatment for lower left extremity osteomyelitis. Our patient faced both intrinsic and extrinsic fall risks. Her intrinsic risks included reduced muscle tone from prolonged care for osteomyelitis and a previous diagnosis of congestive heart failure, which made it difficult to properly exercise. This exacerbated the patient's obesity, which in turn made ambulation more difficult. She also faced extrinsic risks, including the stairs she needed to climb to reach her home. Physician understanding of the nature and risk factors of falls in the elderly can help guide patient education to reduce injuries and situations where a patient may be required to seek placement in a long-term care facility.

## Conclusions

Hematomas can arise from a variety of medications, such as anticoagulants, as emphasized in this case report. While most hematomas may appear harmless, the potential for patients to develop severe complications cannot be underestimated. Our case report underscores the intricate challenges faced by patients with complex medical histories, obesity, and limited mobility, emphasizing the imperative for vigilant monitoring and timely intervention in such cases. The associated risks of anticoagulant use further underscore the necessity for patient education regarding hematoma risks and the importance of prompt symptom reporting. This case report serves as a reminder of the intricacies involved in managing hematomas in high-risk patients, advocating for a multidisciplinary approach, close observation, and proactive patient education to effectively mitigate potential complications. 
